# A Low Noise CMOS Readout Based on a Polymer-Coated SAW Array for Miniature Electronic Nose

**DOI:** 10.3390/s16111777

**Published:** 2016-10-25

**Authors:** Cheng-Chun Wu, Szu-Chieh Liu, Shih-Wen Chiu, Kea-Tiong Tang

**Affiliations:** Department of Electrical Engineering, National Tsing Hua University/No. 101, Sec. 2, Kuang-Fu Road, Hsinchu 30013, Taiwan; clarence1222@gmail.com (C.-C.W.); qoo210709@hotmail.com (S.-C.L.); swchiu1984@gmail.com (S.-W.C.)

**Keywords:** low noise, low power, miniature electronic nose, surface acoustic wave sensor array

## Abstract

An electronic nose (E-Nose) is one of the applications for surface acoustic wave (SAW) sensors. In this paper, we present a low-noise complementary metal–oxide–semiconductor (CMOS) readout application-specific integrated circuit (ASIC) based on an SAW sensor array for achieving a miniature E-Nose. The center frequency of the SAW sensors was measured to be approximately 114 MHz. Because of interference between the sensors, we designed a low-noise CMOS frequency readout circuit to enable the SAW sensor to obtain frequency variation. The proposed circuit was fabricated in Taiwan Semiconductor Manufacturing Company (TSMC) 0.18 μm 1P6M CMOS process technology. The total chip size was nearly 1203 × 1203 μm^2^. The chip was operated at a supply voltage of 1 V for a digital circuit and 1.8 V for an analog circuit. The least measurable difference between frequencies was 4 Hz. The detection limit of the system, when estimated using methanol and ethanol, was 0.1 ppm. Their linearity was in the range of 0.1 to 26,000 ppm. The power consumption levels of the analog and digital circuits were 1.742 mW and 761 μW, respectively.

## 1. Introduction

Electronic nose (E-Nose) systems, which are biomimetic olfactory systems for gas sensing [1], have been used in many applications such as food product quality control [2], indoor air quality monitoring, environmental monitoring [3,4], automotive industry production, and clinical diagnosis [5,6]. These applications involve a steady demand for a portable E-Nose system. One of the reasons for the large size of an E-Nose system is the complexity of the readout circuit [7,8]. Therefore, implementing a readout circuit with an application-specific integrated circuit (ASIC) reduces the size and power consumption of the system and facilitates moving toward a portable E-Nose system.

Surface acoustic wave (SAW)-based gas sensors have the advantages of high sensitivity and fast response [9,10]. Readout circuits for SAW sensors have been developed for years; however, the corresponding power consumption is high and the data resolution is not sufficient for detecting gases at low concentration [11,12]. In our previous work, we implemented a noncontinuous-type readout circuit with integrated circuits (ICs) [10]. We successfully reduced the power consumption to 1.48 mW and improved the resolution to 10 Hz. However, all the sensors in the array did not operate continuously, and a time interval thus existed between the two data points in the measurement results of the same sensor; consequently, data integrity was incomplete.

To improve the data integrity, we propose a continuous-type readout circuit realized by integrating the readout circuit and a cross-coupled pair into an ASIC. By downmixing the operating sensor frequency with a reference sensor frequency, we could further reduce power consumption. Moreover, using a deep N-well technique and modifying the switch timing of the multiplexer enabled us to reduce the noise level of the readout circuit.

In general, an E-Nose system comprises three parts, namely sensor arrays, readout circuits for data acquisition, and data analysis and processing components. The present work focused on implementing a sensor array and its readout circuit. Several sensor types are used for E-Nose systems [13]. Because the primary function of a sensor is to sense an external stimulus, such as detection of a low concentration of gas, the sensor sensitivity is an essential consideration. In general, frequency-type sensors have the advantage of high sensitivity [14]; therefore, in the current work, we applied an SAW sensor to our E-Nose system. However, interference from certain environmental factors is more obvious [15]. To this end, a reference sensor was included to generate a frequency that is least affected by environmental factors.

The SAW resonator structure was composed of a lithium niobate (LiNbO_3_) substrate and aurum (Au) interdigital transducers (IDTs). Various polymer materials were then coated onto the sensor surface to form the sensor array [16]. Studies have reported that coating polymers on sensors can improve the sensitivity of the sensors [13,17], and that the resulting sensor array can facilitate identifying types of gases [18].

In complementary metal oxide semiconductor (CMOS) process technology, substrate noise is an essential consideration in IC design [19]. In a sensor array, the oscillator frequencies of all sensors are extremely close such that injecting current through the ASIC substrate significantly affects the performance of the oscillator output signal. As the number of SAW sensors increases, applying a higher substrate injection current among oscillators raises the output noise level of the SAW sensors. Therefore, we used the deep N-well technique to reduce the interference among different sensors.

For data collection, we implemented a continuous-type interface circuit. Currently, measuring instruments are typically expensive and not portable; therefore, the interface circuit was implemented using an ASIC to achieve reduced size, power consumption, and cost. The interface circuit can convert an analog signal to a digital signal, and it comprises a mixer, low-pass filter, multiplexer, counter, and parallel-to-serial circuit. To compress the interference between channels, we designed a low-noise analog multiplexer to switch the signal from each SAW sensor. 

## 2. Proposed SAW Array and Its Interface ASIC

**Figure 1:**
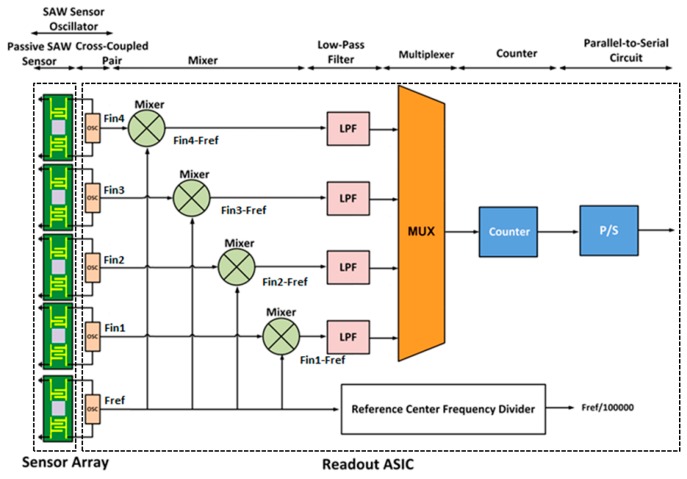
Surface acoustic wave (SAW) array and its interface application-specific integrated circuit (ASIC) block diagram.

Figure 1 outlines a block diagram of the proposed E-Nose structure, which consists of two major parts: the SAW sensor oscillator array and its readout ASIC. The SAW sensor oscillator is composed of a passive SAW sensor and a cross-coupled pair.

The SAW resonator structure is composed of a LiNbO_3_ substrate and Au IDTs. The center frequency of the SAW sensor was determined to be approximately 114 MHz [10]. The surface of the piezoelectric substrate was coated with a thin film of polymer, and the size of SAW sensor is 14 × 7.4 mm^2^.

The sensing mechanism is outlined as follows: when a gas sample is adsorbed on the substrate surface, the change of mass would cause a frequency shift. The sensor array comprises five sensors. Four of them were coated with different polymers, namely poly-N-vinylpyrrolidone, poly-4-vinylphenol, polystyrene, and polystyrene-co-maleic anhydride; the remaining sensor was not coated with any polymer, thus serving as a reference. 

### 2.1. CMOS Cross-Coupled Pair

**Figure 2:**
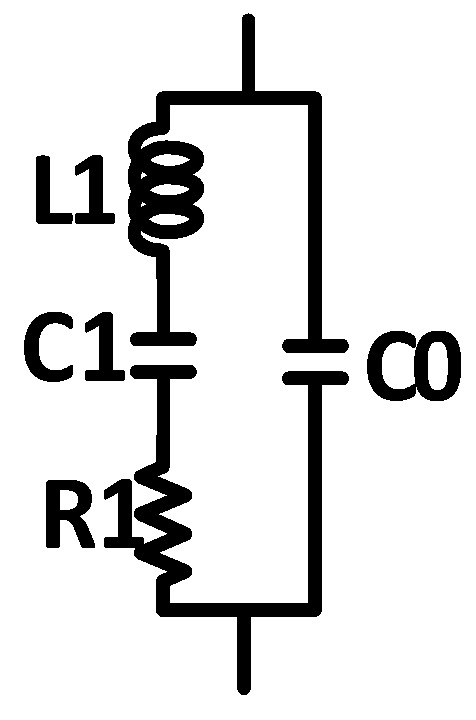
Equivalent circuit model of the SAW sensor.

The sensor of the SAW resonator can be represented by an equivalent circuit model, as depicted in Figure 2. In the model, the inductance, L1, and capacitance, C1, values determine the center frequency of the SAW resonator. Moreover, the parasitic capacitance, C0, and parasitic resistance, R1, must be considered.

**Figure 3:**
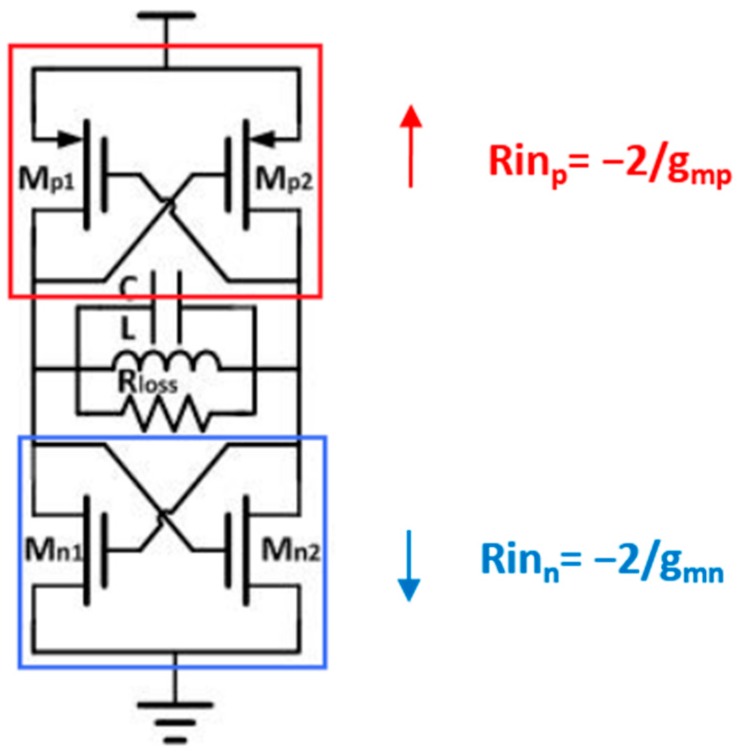
Complementary metal oxide semiconductor (CMOS) cross-coupled pair.

R1 is closely related to the energy loss of the resonator. To reduce this loss, a CMOS cross-coupled pair was attached to introduce a negative resistance with the objective of eliminating the influence of the parasitic resistance. The structure of the CMOS cross-coupled pair is illustrated in Figure 3. 

In the equivalent small-signal model, the input resistance, *R_in_*, between the drain and the source of the metal oxide semiconductor (MOS) can be represented as Equation (1), where all transconductance values are assumed to be *g_m_*:

(1) $$R_{in}=\frac{-2}{g_{m}}$$

The total equivalent resistance, *R_C_*, can then be calculated from Equation (2):

(2) $$R_{c}=(Rin_{p}//Rin_{n})=(\frac{-2}{g_{mp}})//(\frac{-2}{g_{mn}})=\frac{-2}{g_{mp}+g_{mn}}$$

According to Equation (2), *g_mp_* and *g_mn_* are the transconductance values of a p-channel metal oxide semiconductor and an n-channel metal oxide semiconductor (NMOS), respectively. When a gas passes through the SAW sensor, the frequency of the oscillator output shifts. To improve gas detection sensitivity, the noise from the interface circuit should be as low as possible; thus, noise quantification is highly crucial. The noise can be equivalent to another frequency deviation (also called jitter) in the time domain. In the frequency domain, the noise-induced frequency deviation can be transferred to phase noise. Therefore, the phase noise is the most essential design parameter for an oscillator. In the present design, determining the relationship between the phase noise and frequency deviation is vital. Equation (3) shows the phase noise–jitter relationship [20]:

(3) $$J_{RMS}=\frac{1}{2\pi f_{{}_{0}}}\sqrt{2\cdot \int_{f_{1}}^{f_{2}}10^{\frac{L(f)}{10}}df}$$

where *J_RMS_* represents the RMS jitter value (*f_s_*), *f*_0_ represents the center frequency (Hz), *L*(*f*) represents the phase noise magnitude (dBc/Hz), and the integral range is from *f*_1_ to *f*_2_ in the frequency domain. According to the equation, the relationship between the jitter and phase noise is square. Equation (4) shows the period of the oscillator with jitter, where *T′* represents the oscillator period with noise jitter, *f*_0_ represents the oscillator center frequency, and Δ*f* represents the frequency deviation of the oscillator when jitter occurs; notably, the period is not a static value in a period range. According to Equation (5), the RMS jitter is the period deviation Δ*T*, defined as the difference between the maximum and minimum periods, as indicated in Equation (6). Finally, Equation (7) shows the relationship between the frequency deviation and the RMS jitter.

(4) $$\frac{1}{f_{0}+\Delta f}<T\prime <\frac{1}{f_{0}-\Delta f}$$

(5) $$J_{RMS}=\Delta \;T$$

(6) $$J_{RMS}=\frac{1}{f_{0}-\Delta f}-\frac{1}{f_{0}+\Delta f}$$

(7) $$J_{RMS}=\frac{2\Delta f}{{f_{0}}^{2}-\Delta f^{2}}$$

Combining Equations (3) and (7) yields Equation (8), and simplifying Equation (8) yields Equation (9), where the frequency deviation and the phase noise also have a square relationship. On the basis of Equation (9), we can estimate the frequency deviation and calculate the phase noise value in this SAW sensor oscillator to suppress the noise.

(8) $$\frac{2\Delta f}{{f_{0}}^{2}-\Delta f^{2}}=\frac{1}{2\pi f_{{}_{0}}}\sqrt{2\cdot \int_{f_{1}}^{f_{2}}10^{\frac{L(f)}{10}}df}$$

$$\text{Let }M=2\cdot \int_{f_{1}}^{f_{2}}10^{\frac{L(f)}{10}}df$$

(9) $$\Delta f=\frac{\frac{-2}{\sqrt{M}}\pm \sqrt{{\left(\frac{-2}{\sqrt{M}}\right)}^{2}+4\cdot {f_{0}}^{2}}}{2}$$

In this study, when the sensor was in steady-state operation [21], the frequency variation was approximately 20 Hz. Therefore, we set Δ*f* as 20 Hz. Moreover, when *f*_0_ = 115 MHz, the phase noise was less than −177 dBc/Hz at 1 MHz. The overall specifications of the SAW sensor oscillator are presented in Table 1.

### 2.2. Low-Substrate-Noise SAW Sensor Oscillator Array

**Figure 4:**
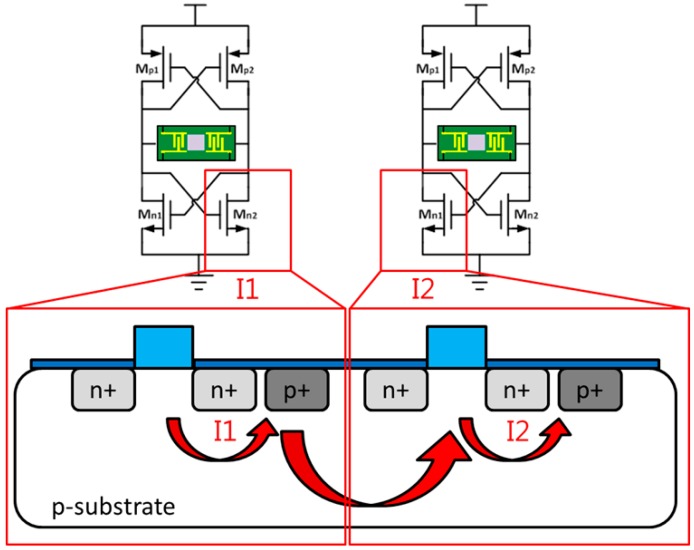
Substrate noise interference between oscillators.

The most critical problem in continuous-type SAW sensor interface circuits is the interference between SAW sensors. Integrating more oscillators on the same chip introduces more interference. The noise affects each oscillator through the substrate [22], and the substrate noise increases the phase noise level associated with the SAW sensor oscillator output frequency. The same substrate was applied for all NMOSs, as shown in Figure 4. One study [23] reported that the occurrence of contact between the back gate and p-substrate engenders interference between oscillators. In the current work, a CMOS cross-coupled pair was used in the oscillator structure.

Equations (10) and (11) show the current of each oscillator. Notably, these equations consist of various amplitudes and frequencies. The summation of these two currents is shown in Equation (12), which can be further simplified to form Equation (13).

(10) $$I_{1}=A\cos(\omega_{1}t+\theta )$$

(11) $$I_{2}=B\cos(\omega_{2}t+\varphi )$$

(12) $$I_{1}+I_{2}=A\cos(\omega_{1}t+\theta )+B\cos(\omega_{2}t+\varphi )$$

(13) I1+I2=ARe{eiθ⋅eiω1t}+BRe{eiφ⋅eiω2t}

To simplify the equation, we assume that A = B = K, and θ = φ θ_K_:

(14) $$I_{1}+I_{2}=2K\cos(\omega_{\Delta }t)\cdot \cos(\omega_{c}t+\theta_{K})$$

$$\text{where }\omega_{c}=\frac{\omega_{1}+\omega_{2}}{2},\omega_{\Delta }=\frac{\omega_{1}-\omega_{2}}{2}$$

From Equation (14), the current sum includes both the frequency sum and difference. This equation demonstrates that a high number of oscillators can cause interference in the same substrate. Moreover, when the current flowing through the substrate is blocked, the interference between the oscillators can be reduced.

**Figure 5:**
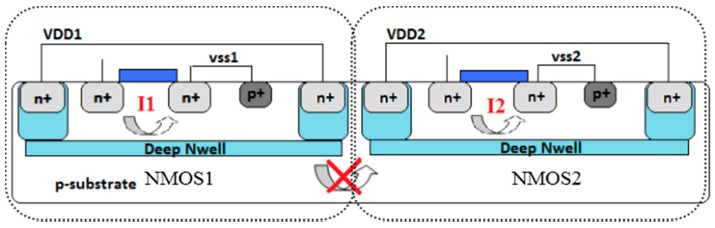
N-channel metal oxide semiconductor (NMOS) deep N-well technique.

**Figure 6:**
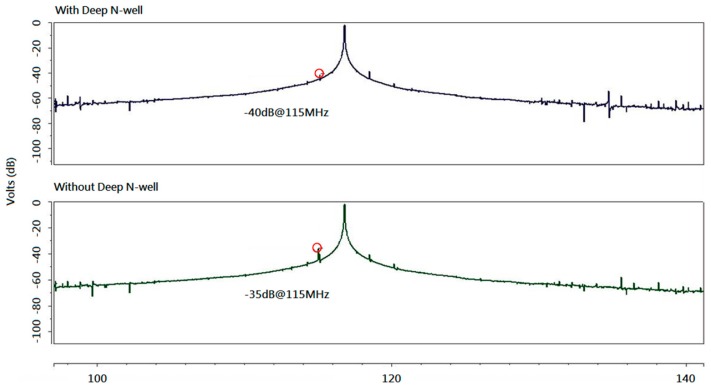
Postlayout simulation of the SAW sensor output spectrum.

To eliminate the interference from the substrate, the deep N-well technique for the NMOS was adopted, as shown in Figure 5 [24,25]. The noise interference between oscillators could be reduced by approximately 5 dB through this method, as depicted in Figure 6.

### 2.3. Mixer and Low-Pass Filter

Environmental factors cause the frequency shift of SAW sensor oscillators. In our design, we included a reference SAW sensor in the SAW sensor array. The reference SAW sensor was not coated, thus generating a frequency with less gas absorption; specifically, this sensor represents the inherent response of the SAW structure to the environment. The center frequency of the reference SAW sensor is highly close to those of the four other coated SAW sensors. Therefore, we can use a mixer to generate a much lower frequency, *f_ref_* − *f_in_*, to simplify the circuit design and reduce the power consumption. We thus applied the reference sensor to generate a frequency, as illustrated in Figure 1.

Before gas entry, we assumed the center frequency of a coated SAW sensor to be *f_in_* and that of the reference SAW sensor to be *f*_0_. After gas entry, the center frequency of the coated SAW sensor was assumed to be *f_in_′*, whereas that of the reference SAW sensor was assumed to be *f_ref_′*. As Equation (15) indicates, the gas-induced frequency change can be derived by subtracting the unwanted frequency, where Δ*f_ref_* (i.e., *f_ref_* − *f_ref_′*) represents the frequency change by environmental factors. After rearrangement, Equation (17) is obtained.

(15) $$\Delta f_{gas}=\Delta f_{ref}-\Delta f_{in}$$

(16) $$\Delta f_{gas}=(f_{ref}\;-\;f_{ref}^{\prime })-(f_{in}-\;f_{in}^{\prime })$$

(17) $$\Delta f_{gas}=(f_{ref}\;-\;f_{in})-(f_{ref}^{\prime }\;-\;f_{in}^{\prime })$$

**Figure 7:**
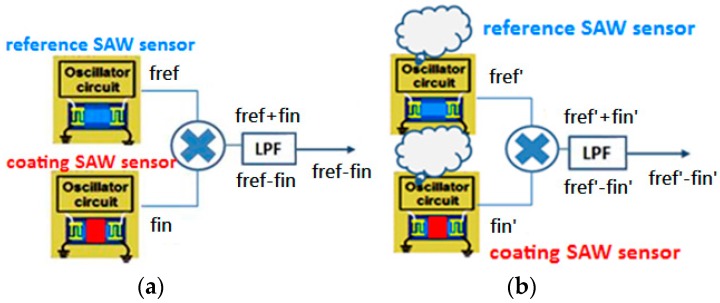
Frequency mixer and low-pass filter (**a**) before the gas enters; and (**b**) after the gas enters.

From Equations (15)–(17), calculating the frequency difference between the coated and the reference SAW sensors yields Δ*f_gas_*. According to the design, a mixer was adopted to obtain both the frequency summation and the frequency difference. Furthermore, a low-pass filter was applied to remove the low-frequency part, as shown in Figure 7.

### 2.4. Multiplexer

In the continuous-type SAW sensor array interface, when the number of sensors on the sensor array increased, the hardware of the total interface circuit became more complex. To reduce the complexity, an analog multiplexer was designed to switch the multichannels. 

To compress the channel switching interference, a low-noise 2-to-1 structure was proposed [26]. When the multiplexer switches to the first channel, no path affects the output node from the second channel. The low-noise structure can change the input MOSFET position of clock signals. To extend the structure, a four-channel multiplexer was implemented. Moreover, to reduce the number of control clocks, we altered the fully NMOS structure into a CMOS structure.

**Figure 8:**
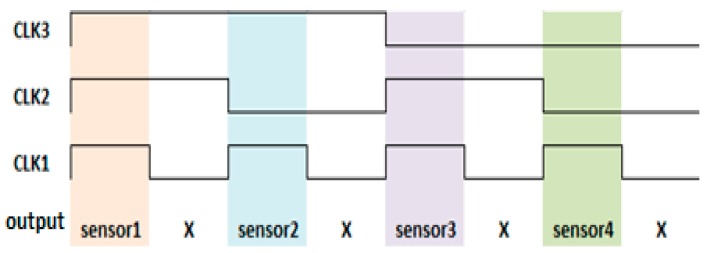
Control clock of the eight-channel multiplexer.

The switch timing of the multiplexer was also modified. Without such modification, the timing of the turned-off MOS and that of the turned-on MOS engender overlapping when the channel switches and cause severe channel interference. To avoid this overlap, the four-channel multiplexer was applied to switch two input signals. Unless the first channel was completely closed, the second channel did not open. To expand to a four-channel system, an eight-channel multiplexer was then applied. The relationship between the control clocks and output channels is outlined in Figure 8.

### 2.5. Time-to-Digital Circuit and Reference Center Frequency Divider

**Figure 9:**
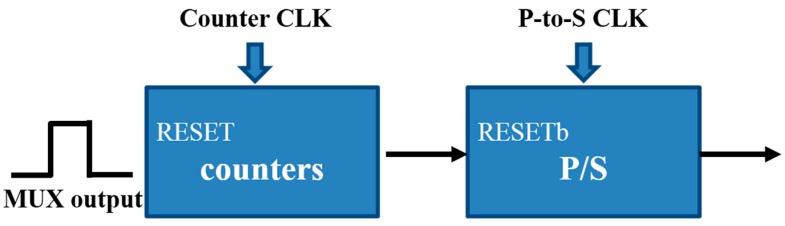
Block diagram of the transferred signal from multiplexer output to digital signal.

Frequency is the reciprocal of time. To derive the time, it can be converted to a frequency signal. For this purpose, a time-to-digital circuit was designed, which included a frequency counter and a parallel-to-serial circuit. The counter transfers *f_in_* − *f_ref_* to a digital signal from the multiplexer output, as depicted in Figure 9.

According to the measurements, the frequency variation was determined to be less than 1 MHz; specifically, the least coverage of measurable frequency was 1 MHz. Therefore, we set the clock frequency as 1 MHz. From Equation (18), the least significant bit of the counter indicates that the least measurable half period is 0.5 μs:

(18) $$f_{0}=\frac{1}{2T_{0}}$$

We set the least difference between frequencies to less than 5 Hz, signifying that the maximum period of the input was 200,000. The half periods of the input can be represented as 11,000,011,010,100,000 in binary format, comprising 17 bits. Therefore, we set 17 as the counter bits. The least measurable difference between the frequencies is 3.8 Hz.

Next, the parallel outputs were converted to serial data. The parallel-to-serial circuit is a shift register, and the AND gate was applied to reduce switching power. 

Restricted by the data processing speed, the center frequency of the signal should be reduced; thus, a divider was designed. Not all the frequency signals could be divided by an integer; therefore, a counting error probably occurred after the reduction of the frequency. A four-stage MOD-10 circuit was implemented to reduce the error because the input number can shift right by 1 bit with every division.

## 3. Experimental Results and Discussion

We used the frequency counter to measure the frequency shift of SAW sensors when different gases were injected. When the gas was changed, the frequency shift also changed.

**Figure 10:**
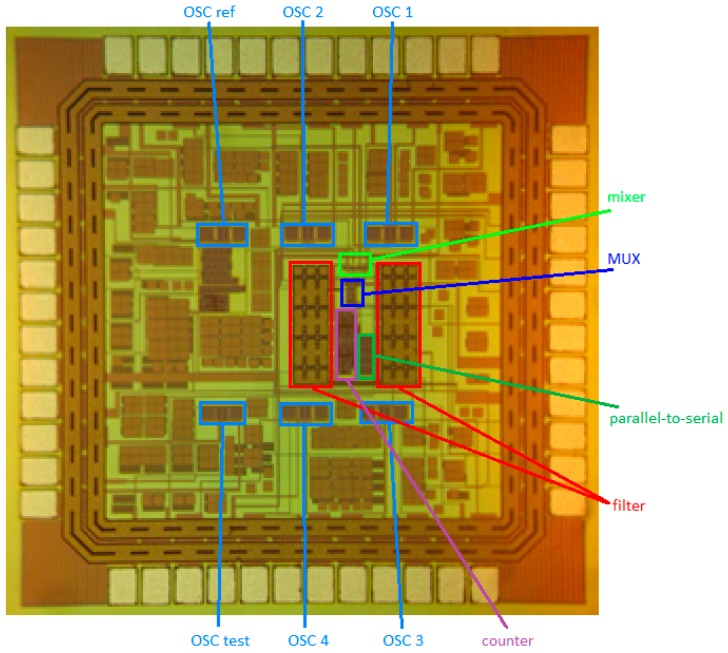
Die photograph of the four-channel continuous-type interface circuit.

The interface circuit was fabricated with TSMC 0.18 μm standard CMOS process. Figure 10 depicts a photograph of a chip die. The total chip size was approximately 1203 × 1203 μm^2^ and the four-channel continuous-type interface circuit comprises four SAW sensor oscillators and one reference SAW sensor oscillator.

**Figure 11:**
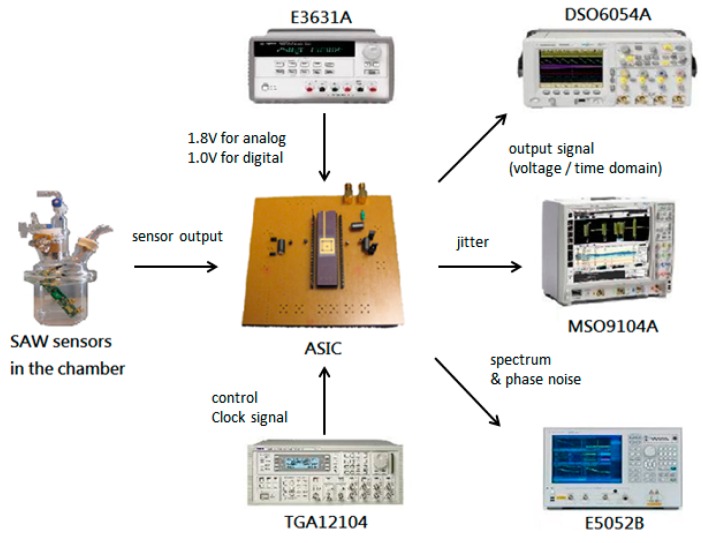
ASIC measurement setup.

Figure 11 illustrates the measurement setup of the ASIC. We used a power supply system (E3631A) to provide 1.8 and 1.0 V to the analog circuit and digital circuit, respectively. The readout ASIC was connected to the SAW sensor array in the chamber, and we used a waveform generator (TGA12104) to transmit the clock control signal to the ASIC. The output signal was sent to an oscilloscope (DSO6054A), a mixed-signal oscilloscope (MSO9104A), and a signal source analyzer (E5052B). Subsequently, we employed the DSO6054A to record the output voltage signal in the time domain, the MSO9104A to measure the jitter of the output signal, and the E5052B to measure the spectra and phase noise.

To test the function of the readout circuit under the gas experiment, we chose methanol and ethanol as the test gases. Methanol and ethanol have similar chemical formulae; therefore, the resolution of the sensor signals must be sufficient to distinguish these two gases.

The gas measurement operations were as follows:

1. Purge the chamber for 5 min.
2. Inject the test gas into the chamber.
3. After 5 min, read the SAW sensor output.
4. Purge the chamber for 5 min.
5. Repeat steps 2–4.

**Figure 12:**
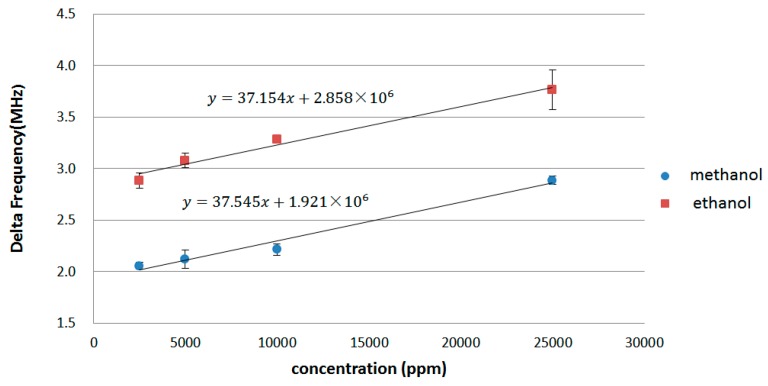
Experimental results for methanol and ethanol gases.

The measurement results for the methanol and ethanol vapors are presented in Figure 12. At the same concentration, the frequency variation of ethanol was greater than that of methanol. In addition, there is a linear relationship between the frequency variation (delta frequency) and gas concentration for both methanol and ethanol. According to [27], the relationship between the frequency shift of SAW sensor and the coated polymer film can be simplified as:

(19) $$\Delta f=(k_{1}+k_{2}){f_{0}}^{2}h\rho$$

where Δf is the frequency shift of SAW sensor, $k_{1}$ and $k_{2}$ are the substrate material constants, $f_{0}$ is the original frequency of SAW sensor, h is the thickness of the film, and ρ is the density of the film. We can assume:

(20) $$h\rho =m_{p}/A$$

where $m_{p}$ is the weight of the polymer film, and A is the sensing area. Thus, the frequency shift of the coated film $\Delta f_{p}$ can be expressed as:

(21) $$\Delta f_{p}=(k_{1}+k_{2}){f_{0}}^{2}m_{p}/A$$

Therefore, the frequency shift caused by the gas adsorption $\Delta f_{gas}$ can be expressed as:

(22) $$\Delta f_{gas}=(k_{1}+k_{2}){f_{0}}^{2}m_{gas}/A$$

where $m_{gas}$ is the weight of the polymer film after gas adsorption. With Equation (22), we can know the frequency variation and the mass variation of the sensor film is in a linear relationship. Therefore, there is a linear relationship between the frequency variation (delta frequency) and gas concentration, as shown in Figure 12. The measurement results suggested that the SAW-based gas sensor worked appropriately for both gases. 

Table 2 outlines the measurement results of the analog and digital circuits. The supply voltage was 1.8 V for the analog part and 1 V for the digital part. The power consumption levels associated with the analog and digital circuits were 1.742 mW and 761 μW, respectively, and the input frequency ranged from 110 to 120 MHz. The detection limit of the system, when estimated using methanol and ethanol, was 0.1 ppm. Their linearity was in the range of 0.1 to 26,000 ppm.

We could derive the sensor output frequency through the SAW interface circuit. Table 3 provides a benchmark for comparing the proposed circuit with previously implemented SAW interface circuits. Overall, we determined the power consumption of the proposed circuit to be lower than those of other circuits.

## 4. Conclusions

This paper presented a low-noise continuous, high-sensitivity CMOS readout circuit for acquiring the signal of an SAW sensor array. The total chip size was approximately 1203 × 1203 μm^2^. The proposed circuit was fabricated in TSMC 0.18 μm 1P6M CMOS process, and the chip was operated at a supply voltage of 1 V for a digital circuit and 1.8 V for an analog circuit.

## Figures and Tables

**Table 1:** Specifications of SAW sensor oscillator.

Parameter	Specifications
Center Frequency (MHz)	110–120
Supply Voltage (V)	1.8
Power (mW)	<10
Phase Noise (1 MHz) (dbc/Hz)	<−177
Frequency Jitter 5 (fs)	<1.5

**Table 2:** Simulations and measurement results of analog and digital circuits.

**Analog**	**Parameter**	**Measurement**
	Supply voltage (V)	1.8
	Phase noise(@1 MHz) (dbc/Hz)	−123.23
	Frequency (Hz)	113.76 M
	Power (W)	1.742 m
	Jitter (s)	36.608 p (1 KHz–10 MHz)
**Digital**	**Parameter**	**Measurement**
	Supply Voltage (V)	1
	Min frequency (Hz)	4
	Max frequency (Hz)	1 M
	Power (μW)	761

**Table 3:** Benchmark of SAW interface circuits.

Parameter	[11]	[12]	[10]	This Work
Supply Voltage (V)	3.3	1.8	3.3	1.0
Process Technology	0.35 μm	0.18 μm	0.18 μm	0.18 μm
Power Consumption (mW)	38.35	4.92	1.48	0.761
Resolution	10 MHz	10 MHz	10 Hz	3.8 Hz
Input Frequency (MHz)	300–400	300–500	117.4	110–120
